# MOVING BEYOND RESEARCH: ARCH AND POLICY

**DOI:** 10.1093/geroni/igae098.1547

**Published:** 2024-12-31

**Authors:** Lauren Brinkley-Rubinstein

**Affiliations:** Duke University, Durham, North Carolina, United States

## Abstract

There is clear evidence that carceral systems negatively impact health, especially for aging populations. The ARCH network has invested in and funded a series of projects that underscore this connection and have clear policy and practice implications. This includes highlighting best practices for compassionate release (both in the context of the COVID-19 pandemic and beyond), creating guidance for the provision of palliative care, optimizing the receipt of correctional health services, outlining the treatment needs of older patients with dementia, and assessing the factors that are integral to implementing a precision-gerontology approach to substance use treatment. In addition, the network has partnered with scholars and practitioners around the country to engage in a series of webinars and “work in progress” meetings to deliver information relevant to best practices, policy guidance, and to brainstorm how best to approach policy change and innovation.

